# PCSK9 levels and diabetic retinopathy: opportunities for a potential target and novel therapeutic approach in conjunction with treating dyslipidaemia

**DOI:** 10.1038/s41433-024-03523-1

**Published:** 2024-12-02

**Authors:** Maria S. Varughese, Ananth U. Nayak, Sarita Jacob

**Affiliations:** 1 University of Sheffield Medical School, Royal Hallamshire Hospital, Sheffield, UK; 2 University Hospitals of North Midlands NHS Trust, Stoke-on-Trent, North Staffordshire UK; 3 School of Medicine, Faculty of Health, Keele University, Newcastle, North Staffordshire UK; 4 University Hospitals Birmingham NHS Foundation Trust, Birmingham, UK; 5 Institute of Clinical Sciences, University of Birmingham, Birmingham, UK; 6 College of Life and Health Sciences, Aston University, Birmingham, UK; 7 Birmingham, Solihull and Black Country Diabetic Eye Screening Programme, Birmingham, UK

**Keywords:** Predictive markers, Clinical pharmacology, Retinal diseases

Atherosclerotic disease is enhanced in people with diabetes, augmenting their cardiovascular risk. Several causes provide likely explanation such as (a) dyslipidaemia, (b) hyperglycaemia, (c) oxidative stress, and (d) inflammation that contribute to the numerous mechanisms within the artery wall [1]. The management of dyslipidaemia, especially with statins per se, have proven to be of enormous benefit in the prevention of clinical cardiovascular disease. However, many patients fail to achieve the low levels of low-density lipoproteins (LDL) recommended in various guidelines, and supplementary therapy, such as the addition of newer proprotein convertase subtilisin/kexin type 9 (PCSK9) inhibitors, are often essential to reach LDL goals [1, 2]. PCSK9 is a secreted protein that is active in the extracellular milieu [3], and it can be neutralised by monoclonal antibodies. PCSK9 is found in chromosome-1 and mutation in that specific gene causes familial hypercholesterolemia [4].

PCSK9 plays an instrumental role in cholesterol homeostasis by promoting LDL receptor degradation in the lysosomes of hepatocytes, and inhibition of PCSK9 results in significantly increased number of LDL receptors on the hepatocyte membrane, resulting in greater catabolism of circulating LDL particles [2]. Therefore, blocking the activity of PCSK9 with monoclonal antibodies decreases the degradation of LDL receptors, increases the clearance of LDL-cholesterol (LDL-C), and an injection of PCSK9-specific antibody suppresses LDL-C concentrations for several weeks [3]. There are two PCSK9 inhibiting monoclonal antibodies, alirocumab and evolocumab, administered every 2–4 weeks that were approved for treating dyslipidaemia and they have been effective lipid-lowering drugs with greater absolute risk-reduction for cardiovascular events particularly in patients with diabetes, decreasing LDL-C by 50–65% [2].

PCSK9 inhibitors (PCSK9i) clearly demonstrate LDL-C lowering and is hugely beneficial in patients with familial hypercholesterolemia and for secondary prevention of acute cardiovascular events in high-risk patients with atherosclerotic cardiovascular disease [3–5]. The addition of PCSK9i to statins or other lipid-lowering therapies is not associated with an increased risk of adverse events [5].

In people with diabetes, treatment with the PCSK9i significantly reduced atherogenic lipids with no adverse effects on glycaemic control, and these medications were well tolerated, with the magnitude of the benefit depending largely on the absolute reduction in LDL-C achieved [2, 6, 7]. The ODYSSEY, FOURIER and OSLER study groups have established the valuable outcomes of this group of drugs in achieving significant benefits in the most at-risk cohort of patients [8–11]. More recent smaller studies continue to show the advantages of treating high-risk patients with PCSK9i [12].

Inclisiran is another first-in-class small interfering RNA (siRNA) against PCSK9 that has been approved by both the US Food and Drug Administration (FDA) and the European Medicines Agency (EMA) for the treatment of hypercholesterolemia [13]. Of note, inclisiran treatment facilitated an improvement in LDL-C target achievement by enabling a prolonged and distinct LDL-C lowering effect [13–15].

The ORION-8 trial, provides the largest and longest follow-up data on inclisiran to date demonstrating that twice-yearly administration of inclisiran, subsequent to the initial first dose and next one at month-3 provided consistently effective LDL-C lowering with no new safety signals during an additional mean treatment exposure of 2.6 years beyond the parent trials, with 8530 patient-years of exposure [14, 15]. These findings illustrates the consistent long-term efficacy and tolerability of inclisiran over a sustained period of exposure of 6.8 years [14].

Diabetic atherosclerosis is a complex process that is characterised by diffuse and unstable lesions increasing 2-4-fold the risk of adverse cardiovascular events, with diabetic dyslipidaemia having a predominant role in coronary artery disease, and the benefits of PCSK9i in reducing this risk must be reiterated [16–18].

In the context of diabetic retinopathy (DR), it is interesting to note that PCSK9 levels have been positively co-related in patients with diabetes, increasing in patients with progressing grades of DR compared to controls with no diabetes [19]. This recent study with small numbers explored four groups of patients and the mean PCSK9 level in each of these cohorts [*group 1* (diabetes and no DR): 0.24 ± 0.09 ng/mL, *group 2* (diabetes and non-proliferative DR): 0.29 ± 0.08 ng/mL, *group 3* (diabetes and proliferative DR): 0.38 ± 0.11 ng/mL, *group 4* (matched controls with no diabetes): 0.13 ± 0.07 ng/mL]. The PCSK9 values between the groups showed a statistically significant difference (*p* < 0.001), with the highest values in *group 3*, and an increasing trend of these values were observed with progression of DR. When serum PCSK9 levels were evaluated in the diabetic patients (all in groups 1, 2 and 3, *n* = 100) compared to the control group 4 (people with no diabetes), these were found to be significantly higher in diabetic patients (*p* = 0.001), and highest in group 3. In addition, when the serum PCSK9 levels were evaluated in all diabetic patients, a medium-level correlation with LDL was observed (*p* < 0.05).

When the groups were evaluated separately, no significant correlation was observed with the levels of high-density lipoprotein (HDL) and triglycerides. Their analysis revealed that there was no significant association between serum PCSK9 levels and patients’ age, when all groups were analysed separately or together (*p* > 0.05), and again no significant correlation was found between HbA1c and serum PCSK9 levels between these groups [19].

It is also pertinent to observe that emerging evidence from recent studies with PCSK9i treatment in the context of lipid lowering in patients with diabetes, has also been shown to have a reduced risk of progressing to secondary complications of DR [20], indicative of a retino-protective effect.

The PCSK9i evolocumab, has been reported to have a protective effect against inflammation in rat retinal Muller cells (RMCs), at least partially, by negatively regulating the activation of the TLR-4/NF-κB signalling pathway [21]. Hence, evolocumab could consequently be a potentially feasible anti-inflammatory therapy for DR. RMCs are the predominant sources of various inflammatory factors that control the inflammatory response within the retina. Therefore, RMCs are most likely, the dominant glial cells in the entire retina. In the early stages of DR, RMCs endure changes much before endothelial cells and pericytes [21], underpinning the need to find drugs that inhibit RMC activation and the ensuing production of proinflammatory cytokines.

A Mendelian Randomization study has offered evidence of a positive association between PCSK9-mediated LDL-C levels and DR risk at a genetic level [22]. The elevated PCSK9-mediated LDLC levels was understood to be related with a heightened risk of DR, hypothesising the feasibility of a causal relationship between PCSK9 inhibition and a reduced risk of DR. Interestingly, none of the HDL and triglyceride targets were related to DR risk, signifying that modulation of the LDL-C target [19, 20], rather than the TG or HDL target [21, 22], could reduce the risk of DR mediated by PCSK9.

Indeed, there is now a renewed interest in reducing the progression of DR, in conjunction with lipid modifying treatment, especially in view of the results of the recent LENS trial [23, 24]. These resonate not only with the previous FIELD and ACCORD-EYE studies related to fenofibrate therapy [25], but is certainly also relevant to treating dyslipidaemia with PCSK9i which could pave the way for opportunities towards a novel therapeutic approach to managing DR. Further randomised controlled trials (RCTs) are warranted to validate DR correlation with PCSK9i therapy.

Patients with diabetes and primary familial hypercholesterolaemia or non-familial mixed dyslipidaemia will be eligible to be recruited to future RCTs (Alirocumab and Evolocumab when LDL-C is ≥3.5 mmol/l and Inclisiran for secondary prevention with LDL-C of ≥2.6 mmol/l).

## References

[1] Chait A, Eckel RH, Vrablik M, Zambon A. Lipid-lowering in diabetes: An update. Atherosclerosis. 2024;394:117313. 10.1016/j.atherosclerosis.2023.117313. 37945448 10.1016/j.atherosclerosis.2023.117313

[2] Béliard S, Mourre F, Valéro R. Hyperlipidaemia in diabetes: are there particular considerations for next-generation therapies? Diabetologia 2024;67:974–84. 38376536 10.1007/s00125-024-06100-z PMC11058750

[3] Giglio RV, Muzurović EM, Patti AM, Toth PP, Agarwal MA, Almahmeed W, et al. Treatment with Proprotein Convertase Subtilisin/Kexin Type 9 Inhibitors (PCSK9i): Current Evidence for Expanding the Paradigm? J Cardiovasc Pharm Ther. 2023;28:10742484231186855. 10.1177/10742484231186855 37448204

[4] Hajar R. PCSK 9 Inhibitors: A Short History and a New Era of Lipid-lowering Therapy. Heart Views. 2019;20:74–5. 31462965 10.4103/HEARTVIEWS.HEARTVIEWS_59_19 PMC6686613

[5] Choi HD, Kim JH. An Updated Meta-Analysis for Safety Evaluation of Alirocumab and Evolocumab as PCSK9 Inhibitors. Cardiovasc Ther. 2023;2023:7362551. 36704607 10.1155/2023/7362551 PMC9834631

[6] Monami M, Sesti G, Mannucci E. PCSK9 inhibitor therapy: A systematic review and metaanalysis of metabolic and cardiovascular outcomes in patients with diabetes. Diabetes Obes Metab. 2019;21:903–8. 30485622 10.1111/dom.13599

[7] Chen T, Wang Z, Xie J, Xiao S, Li W, Liu N. Efficacy and safety of PCSK9 inhibitors in patients with diabetes: A systematic review and meta-analysis. Nutr Metab Cardiovasc Dis. 2023;33:1647–61. 37414664 10.1016/j.numecd.2023.05.033

[8] Robinson JG, Farnier M, Krempf M, Bergeron J, Luc G, Averna M, et al. Efficacy and safety of alirocumab in reducing lipids and cardiovascular events. N Engl J Med. 2015;372:1489–99. 25773378 10.1056/NEJMoa1501031

[9] Bittner VA, Schwartz GG, Bhatt DL, Chua T, De Silva HA, Diaz R, et al. Alirocumab and cardiovascular outcomes according to sex and lipoprotein(a) after acute coronary syndrome: a report from the ODYSSEY OUTCOMES study. J Clin Lipido. 2024;18:e548–e561. 10.1016/j.jacl.2024.04.122 38960812

[10] Sabatine MS, Giugliano RP, Keech AC, Honarpour N, Wiviott SD, Murphy SA, et al. Evolocumab and Clinical Outcomes in Patients with Cardiovascular Disease. N Engl J Med. 2017;376:1713–22. 28304224 10.1056/NEJMoa1615664

[11] Sabatine MS, Giugliano RP, Wiviott SD, Raal FJ, Blom DJ, Robinson J, et al. Efficacy and safety of evolocumab in reducing lipids and cardiovascular events. N Engl J Med. 2015;372:1500–9. 25773607 10.1056/NEJMoa1500858

[12] Laufs U, Birkenfeld AL, Fraass U, Hohenstein B, Siegert C, Klotsche J, et al. Novel Insights into the Management of Patients with Very High Cardiovascular Risk Eligible for PCSK9 Inhibitor Treatment: Baseline Findings from the PERI-DYS Study. Cardiovasc Drugs Ther. 2024;38:119–29. 36178485 10.1007/s10557-022-07386-0 PMC10876819

[13] Katsiki N, Vrablik M, Banach M, Gouni-Berthold I. Inclisiran, Low-Density Lipoprotein Cholesterol and Lipoprotein (a). Pharmaceuticals. 2023;16:577. 37111334 10.3390/ph16040577 PMC10143414

[14] Wright RS, Raal FJ, Koenig W, Landmesser U, Leiter LA, Vikarunnessa S, et al. Inclisiran administration potently and durably lowers LDL-C over an extended-term follow-up: the ORION-8 trial. Cardiovasc Res. 2024;120:1400–10. 38753448 10.1093/cvr/cvae109 PMC11481169

[15] Ray KK, Troquay RPT, Visseren FLJ, Leiter LA, Scott Wright R, Vikarunnessa S, et al. Longterm efficacy and safety of inclisiran in patients with high cardiovascular risk and elevated LDL cholesterol (ORION-3): results from the 4-year open-label extension of the ORION-1 trial. Lancet Diabetes Endocrinol. 2023;11:109–19. 36620965 10.1016/S2213-8587(22)00353-9

[16] Velidakis N, Stachteas P, Gkougkoudi E, Papadopoulos C, Kadoglou NPE. Classical and Novel Lipid-Lowering Therapies for Diabetic Patients with Established Coronary Artery Disease or High Risk of Coronary Artery Disease-A Narrative Clinical Review. Pharmaceuticals. 2024;17:568. 38794138 10.3390/ph17050568 PMC11124492

[17] Imbalzano E, Ilardi F, Orlando L, Pintaudi B, Savarese G, Rosano G. The efficacy of PCSK9 inhibitors on major cardiovascular events and lipid profile in patients with diabetes: a systematic review and meta-analysis of randomized controlled trials. Eur Heart J Cardiovasc Pharmacother. 2023;9:318–27. 36972610 10.1093/ehjcvp/pvad019

[18] Chen T, Liu N. How safe are proprotein convertase subtilisinekexin type 9 inhibitors in diabetes? Curr Opin Lipido. 2024;35:187–94. 10.1097/MOL.0000000000000934 38527426

[19] Karapapak M, Kara ZMY, Düzgün E. The Predictive Utility of Circulating PCSK9 Levels on Diabetic Retinopathy Stage. Curr Eye Res. 2024;49:1107–13. 39086188 10.1080/02713683.2024.2386360

[20] Jeong H, Shaia JK, Talcott KE, Singh RP. Investigating the Relationship Between LipidLowering Agents and the Complications of Diabetic Retinopathy. Ophthalmic Surg Lasers Imaging Retina. 2024:1–8. 10.3928/23258160-20240729-03. 10.3928/23258160-20240729-03 39231114

[21] Zhou Q, Tang H, Li S. Protective effect of evolocumab on Müller cells in the rat retina under hyperglycaemic and hypoxic conditions. J Diabetes Complications. 2023;37:108593. 37717351 10.1016/j.jdiacomp.2023.108593

[22] Chen S, Zhang M, Yang P, Guo J, Liu L, Yang Z, et al. Genetic Association between LipidRegulating Drug Targets and Diabetic Retinopathy: A Drug Target Mendelian Randomization Study. J Lipids. 2024;2024:5324127. 38757060 10.1155/2024/5324127 PMC11098603

[23] Preiss D, Logue J, Sammons E, Zayed M, Emberson J, Wade R, et al. Effect of Fenofibrate on Progression of Diabetic Retinopathy. NEJM Evid. 2024;3:EVIDoa2400179. 38905569 10.1056/EVIDoa2400179 PMC7616293

[24] Silva PS, Aiello LP. Fenofibrate Shows Promise in Slowing Diabetic Retinopathy Progression. NEJM Evid. 2024;3:EVIDe2400205. 39041872 10.1056/EVIDe2400205

[25] Varughese MS, Nayak AU, Jacob S. Fenofibrate therapy in reducing the progression of diabetic retinopathy: revisiting the FIELD and ACCORD-EYE studies through the LENS trial. Eye. 2024. 10.1038/s41433-024-03410-9. 10.1038/s41433-024-03410-9 PMC11733283 39438742

